# Strategies for Recruiting African Immigrants to Mixed Methods Maternal Health Research: Evidence from a Pilot Study

**DOI:** 10.1007/s10903-025-01813-8

**Published:** 2025-11-05

**Authors:** Billan Mahdi, Mary Ghebreselassie, Maraki Solomon, Mouneissa Wangara, Meymuna Hussein-Cattan, Mahader Tamene, Elleni M Hailu, Safyer McKenzie-Sampson

**Affiliations:** 1 Department of Pediatrics, Stanford University, Stanford, United States; 2 African Advocacy Network, San Francisco, United States; 3 Tiyya Foundation, Santa Ana, United States; 4 Division of Epidemiology, University of California, Berkeley, Berkeley, United States; 5 Department of Health Behavior and Health Equity, University of Michigan–Ann Arbor, Ann Arbor, United States

**Keywords:** Maternal health, African immigrants, Black women, Mixed methods, Pregnancy, California

## Abstract

African immigrants in the United States (US) have a lower average risk of adverse birth outcomes, compared to US-born Black women. While population-level studies have identified potential explanatory factors, including differences in exposure to racism and access to social support, there are few mixed methods investigations among African immigrants on the West Coast of the US. In this brief report, we outline the strategies deployed to recruit African immigrant mothers in California to the AZANIA (**A**frican immigrants conceptuali**Z**ing pregn**AN**cy experiences **I**n **A**merica) pilot mixed methods study. The AZANIA study was grounded in community-engaged research principles and aimed at understanding the relationship between racism, social support, and use of African cultural practices during pregnancy and childbirth. Within five months we met 100% (*n* = 25) of our recruitment goal, despite restrictive immigration policies in California. Among the ten recruitment strategies used in the AZANIA study, five effective strategies to recruit African immigrant mothers emerged: referrals from community partner organizations (*n* = 8), snowball sampling (*n* = 5), referrals from personal contacts of African descent (*n* = 5), flyers at African-owned businesses (*n* = 5) and flyers in Black health-focused newsletters (*n* = 2). Future studies should consider our culturally-grounded recruitment strategies in their upcoming mixed methods research among African immigrant mothers in the US.

## Introduction

African immigrants in the United States (US) have a lower average risk of adverse birth outcomes compared to US-born Black women [1, 2]. While population-level investigations have studied social support and reduced exposure to racism as potential factors that explain the difference in risk, there are few mixed methods studies focused on African immigrants in the US to understand these nuanced relationships [3, 4]. We launched the AZANIA (**A**frican immigrants conceptuali**Z**ing pregn**AN**cy experiences **I**n **A**merica) Study in California. The AZANIA study was a statewide mixed methods pilot study from February to June 2025, exploring the ways in which racism, social support, and African cultural traditions influence the pregnancy and childbirth experiences of African immigrants.

African immigrants were eligible for the AZANIA study if they identified as Black, were born in Africa, 18 years of age or older, had a live birth in the US within the last 5 years, and resided in California. AZANIA study participants completed a quantitative survey with 7 validated scales to measure exposure to racism, acculturation, resilience, social support and chronic stress, as well as a 2-hour virtual qualitative interview, receiving a $120 gift card for their time. We grounded our study in the principles of community-based participatory research to include the diverse perspectives of the African immigrant community in California [5]. This included partnering with two trusted African immigrant-led community-based organizations in Northern (African Advocacy Network) and Southern California (Tiyya Foundation) to ensure cultural and community relevance of our research [5]. We also assembled a multilingual team of African descent to provide all recruitment and study materials in English, French, Amharic, Tigrinya and Somali. Study materials were professionally translated by medically certified translators to ensure cultural applicability of terminology. This aligns with the linguistic needs of the African immigrant population in California [1], and encouraged mothers with low English proficiency to join the study.

We employed 10 strategies to recruit women to the AZANIA study. In this brief report, we outline each strategy and provide advice for future researchers interested in recruiting African immigrants for mixed methods maternal health studies.

## Methods

### Strategy 1: Referrals from Community-Based Partner Organizations

We onboarded our aforementioned community-based partner organizations as paid consultants to increase awareness about the study in the African community. These organizations have direct contact with eligible African immigrants in California and were able to answer any questions prospective African mothers had about the study. Our contacts at the organizations would pre-screen potential participants before referring them to the AZANIA study staff for intake.

### Strategy 2: Reaching Out To Personal Contacts of African Descent

The study team reached out to personal contacts including local medical professionals (i.e., doctors, nurses, and research coordinators) and faith leaders of African descent, as they may know of eligible African immigrant mothers within their personal and professional networks. Contacts were encouraged to share the study flyer among their patients, family, and friends who might be either interested in the study themselves or know someone else who was eligible.

### Strategy 3: Flyers at Local African-owned Businesses

We conducted in-person outreach at 11 local African-owned businesses, including restaurants and grocery stores. We visited each location, introduced ourselves and the study, explained its purpose, and built rapport with the store owners and staff. With their permission, we displayed flyers on-site in high-visibility areas such as entrances, check-out counters, and community boards. Additionally, we encouraged staff to share information about the study with their clientele. This strategy cost approximately $500, including the costs of printing flyers in the five study languages, and vehicle rental fees to facilitate travel to the businesses.

### Strategy 4: Snowball Referrals from Previously Enrolled AZANIA Study Participants

We encouraged recruited AZANIA participants to spread the word about the study in their social networks and among friends and family. Snowball sampling can prove useful in recruiting participants who may feel more comfortable joining a study vetted by someone they know [6], especially during times of increased restrictive federal immigration policies.

### Strategy 5: Flyers Shared in Black Health Newsletters

We shared the study flyer among statewide Black health distribution lists and newsletters. These email distribution lists reach health researchers who we hoped would be interested in raising awareness about the study within their networks.

### Strategy 6: Flyers at Local Clinics

Clinical encounters create natural opportunities for providers to mention the AZANIA study to African parents. Thus, we collaborated with a pediatrician and family physician in San Francisco to affix the study flyer in their clinics, and encouraged them to discuss the study with their African immigrant patients during appointments.

### Strategy 7: Attendance at Events for the African Community

The study team attended five events during Black History Month and Women’s History Month hosted by local organizations that support African communities. These events provided the opportunity to share information about the AZANIA study goals and eligibility requirements with influential members of the African community.

### Strategy 8: Direct Messaging To non-partnered African Immigrant Groups in California

To increase our exposure within groups beyond our two community-based partner organizations, we compiled a list of 26 additional organizations that serve African immigrants in California, including faith-based institutions, social groups, and professional networks. While we had no previous contact with these groups, we sent personalized emails to each organization to increase awareness of the study. Four organizations responded and posted the AZANIA study flyer on their social media channels. In addition, the study PI collaborated with a Southern California Nigerian social organization to lead an online informational session on the AZANIA study with potential participants.

### Strategy 9: Social Media

We utilized social media platforms Instagram and Facebook to share study flyers accompanied by helpful information to potential participants. Our social media content included visual summaries of data on African immigrant maternal and child health in California. We also posted stock images of African women and ‘Birth Tradition Thursday’ graphics, where we spotlighted a different African Birth Tradition each week. All posts contained curated hashtags aimed to increase targeted engagement, and we averaged 100 views per post on each platform. We additionally launched an official study website where mothers could express interest in the study.

### Strategy 10: Radio Show

The study PI was an invited guest to Africa Mix on KALW Public Media, a San Francisco Bay Area local radio program. Africa Mix plays music from the African diaspora on a weekly basis. During the segment, the PI shared the study goals and facts about African maternal health with listeners in hopes of recruiting eligible mothers.

## Results

**Fig. 1:**
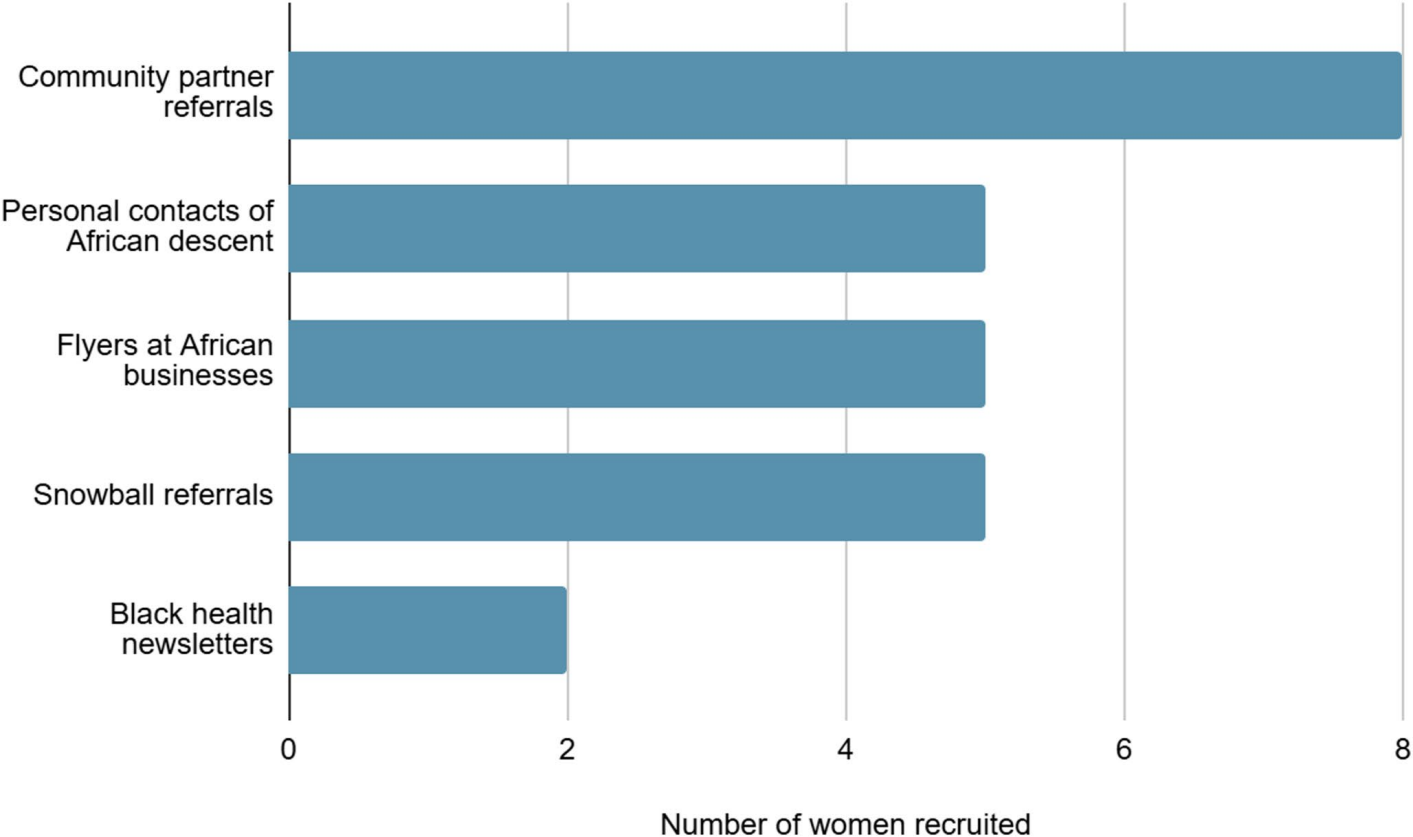
Successful recruitment strategies among African immigrant Black mothers in California

We screened 31 women who inquired about the study, and reached our recruitment goal (*n* = 25) within 5 months. While women could have heard about the study through multiple platforms, every AZANIA study participant mentioned only one recruitment strategy through which they heard about the study. Five of the strategies employed were effective (Fig. 1). Direct referrals from our community partner organizations were the most successful strategy, accounting for 32% (*n* = 8) of participants. Snowball sampling, in-person flyers at African-owned businesses, and referrals from personal contacts of African descent were moderately successful strategies, each leading to 20% (*n* = 5) of our study sample, respectively. The final 8% (*n* = 2) of participants were recruited through Black health newsletters.

## Discussion

The most successful recruitment strategies were culturally grounded and community-driven, leaning on the strengths of organizations that had previously established trust within the African immigrant community in California. The majority of mothers in the study were recruited via our community-based partner organizations, as African mothers were more likely to participate when informed about the study from an organization where they have previously received services. Moreover, our community partners assisted us with reaching a wider audience, as they recruited mothers from countries with low representation in California (e.g., Angola and Togo) [3].

Our results align with previous studies that recruited African immigrants in the US, which found that word of mouth from trusted contacts such as family, friends and community leaders are effective recruitment strategies [7–9]. While other studies found social media to be a powerful recruitment tool among African immigrants [10], our digital outreach was unsuccessful and led to over 300 fake study registrants which forced us to shut down the study website. However, previous studies leveraging existing African immigrant social media messaging groups were successful, whereas we created new social media accounts dedicated to the AZANIA study which did not have an established large following of African immigrants to increase engagement. We also limited recruitment to California, and successful strategies may need to be adapted in other geographic contexts.

## Conclusion

Despite heightened immigration and customs enforcement presence in California in 2025, African immigrant mothers were interested in participating in our maternal health mixed methods study. African mothers were readily recruited from community partner organizations, snowball sampling, personal contacts of African descent, flyers at African-owned businesses and in Black health-focused newsletters. Researchers interested in recruiting African mothers should consider these strategies, and utilize social media with caution so as to filter out non-eligible individuals or scam registrants.

## Data Availability

No datasets were generated or analysed during the current study.
